# Surgical resection for locally invasive renal cell carcinoma: Is it worthwhile?

**DOI:** 10.4103/0970-1591.33444

**Published:** 2007

**Authors:** Suraj Manjunath, C. Ramachandra, Vijayashree Murthy, Prashanth S. Murthy, P. S. Prabhakaran, V. Satya Suresh Attili

**Affiliations:** Department of Surgical Oncology, Kidwai Memorial Institute of Oncology, Karnataka, India; * Department of Medical Oncology, Kidwai Memorial Institute of Oncology, Karnataka, India

**Keywords:** Locally invasive, radical nephrectomy, renal cell carcinoma

## Abstract

**Background:**

Many patients with renal cell carcinoma (RCC) present with disease involving the adjacent viscera. Although survival in such patients is poor, surgery remains the only proven modality of treatment. We describe our experience with radical nephrectomy for locally invasive RCC over a five-year period.

**Study Design:**

A retrospective analysis of the records of all patients who had undergone surgery for locally invasive RCC between January 1999 and December 2004 at our institute.

**Materials and Methods:**

During the study period, 102 patients with RCC underwent surgery at our institute, out of which 18 (17.6%) patients had adjacent organ involvement. The survival and outcomes in terms of symptom relief are described.

**Statistical Analysis:**

The survival rates were calculated by the Kaplan-Meier method using EGRET statistical software package.

**Results:**

Of the 18 patients, two patients had inoperable disease. Fifteen out of the 18 patients succumbed to their disease after a median period of 7.5 months. Three patients are still alive, having survived for 13, 16 and 25 months. Most patients derived considerable benefit with respect to relief of symptoms, which was long-lasting.

**Conclusion:**

For selected patients with locally invasive RCC, radical nephrectomy with en bloc resection of involved organs may provide the opportunity for long-term survival. In others, it may provide considerable symptomatic relief.

Today more and more patients with renal cell carcinoma (RCC) are being detected incidentally at an early stage because of the increasing use of radiological investigations. However, about one-fourth of patients with RCC continue to present with locally advanced tumors[1] and this includes patients with RCC involving adjacent viscera. Tumors extending beyond the Gerota's fascia and involving adjacent organs or structures (other than the ipsilateral adrenal gland) are known as locally invasive tumors. Patients with such tumors have a uniformly poor prognosis[2] and this is reflected in the AJCC TNM staging, which classifies such tumors along with Stage IV disease.[3] Only a small fraction of the vast literature on RCC has been devoted to locally invasive disease, indicating the dismal outlook for these patients.[4] Surgery for massive RCCs involving adjacent viscera is still controversial, because the results of such extensive resection are poor in terms of long-term survival.[5]

RCC can spread locally to infiltrate any structure in the area and involvement of the psoas muscle, colon, mesentery, pancreas, spleen, duodenum and diaphragm have all been described.[6–8] The liver can also be involved by direct extension, but metastatic spread is much more common.[9]

Almost all of these patients are incurable and even with radical surgical excision, less than 5% of patients survive three years after diagnosis.[10]

However, since surgery remains the only effective treatment for such tumors, extended resections may occasionally be warranted. Even though long-term survival is rare, palliation and local control are achievable goals. Resection of all invaded organs is recommended, because debulking or partial resection does not benefit the patient.[10]

## MATERIALS AND METHODS

The present study is based on a retrospective analysis of hospital records of patients undergoing surgery for renal cell carcinoma from January 1999 to December 2004. During the study period, 102 patients underwent surgery for RCC at our institute. Preoperative workup included either ultrasound (USG) or computerized tomography (CT) of the abdomen for evaluation of loco-regional disease and intra-abdominal metastases. Routine blood tests, chest radiograph and, in selected patients, an isotope bone scan were obtained.

Based on intraoperative findings, 18 patients were identified as having locally invasive disease, i.e., disease involving adjacent organs. None of these patients had evidence of distant metastases on preoperative workup and those patients found to have distant metastases intraoperatively (liver, peritoneum, opposite kidney or opposite adrenal gland) were excluded from the study. Radical nephrectomy with en bloc resection of involved organ/organs was performed in all but two of the patients. These two patients were considered inoperable and no procedure was carried out. Following surgery, the patients were assessed regarding relief of their major preoperative symptoms and the duration of such relief. The progression of disease and pattern of recurrence were studied. Survival figures were analyzed and are presented below.

### Statistical analysis

Survival analysis was calculated by the Kaplan-Meier method using EGRET statistical software package. Subset analysis of variables could not be performed because of the small number of patients.

## RESULTS

Of the 18 patients with locally invasive RCC, 13 were male and five female. The age of the patients ranged from 39 to 73 years with a mean of 57.5 years. Operative findings included involvement by the tumor of the spleen (n=5), abdominal wall (n=5), ascending colon with mesocolon (n=2), spleen along with tail of pancreas (n=2) posterior abdominal wall along with diaphragm (n=1), duodenum (n=1), liver and diaphragm (n=1) and abdominal aorta (n=1). Patient and tumor characteristics are shown in Table 1.

**Table 1:** Patient and tumor characteristics

Number of patients	18
Age	39 – 73 years (mean 57.5 years)
Sex	Male: 13, female: 5
Extrarenal organ of	Spleen − 5
involvement	Abdominal wall − 5
Ascending colon with mesocolon − 2	
	Spleen + tail of pancreas − 2
	Posterior abdominal wall + diaphragm − 1
	Duodenum − 1
	Liver + diaphragm − 1
	Abdominal aorta − 1
Fuhrman's grading (16/18)	Grade I − 3, Grade II − 5, Grade III − 6,
	Grade IV − 2

In two patients, no surgical procedure could be performed because of extensive disease, in one case because of infiltration of the abdominal aorta and in the other because of extensive involvement of the liver and diaphragm. The remaining 16 patients underwent radical nephrectomy with en bloc resection of the involved organ/organs.

Histopathology confirmed involvement of extrarenal disease in 13 of 16 patients who underwent resection. In three patients, i.e. two with splenic involvement and one with involvement of the posterior abdominal wall, intraoperative suspicion of organ involvement was not borne out by final histopathology. The grade of the tumor in the resected specimens according to Fuhrman's grading was Grade I in three, Grade II in five, Grade III in six and Grade IV in two patients.

Two patients received postoperative interferon alpha therapy, including one with inoperable disease. The remaining patients could not afford the cost of interferon therapy.

**Figure 1:**
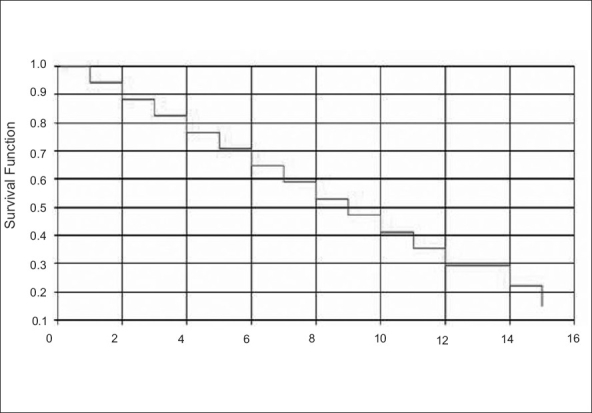
Overall survival in patients with locally invasive RCC

Fifteen of the 18 patients succumbed to their disease after a median period of 7.5 months (range two to 16 months) [Figure 1]. The most common site of disease recurrence was the lung (n=11), all recurrences being multifocal and inoperable. Both the patients with inoperable primary disease were dead within four months of surgery. Three patients are still alive at time of submission of this article, having survived for 13, 16 and 25 months, out of whom one has developed multiple lung metastases. Variables like age, sex, extrarenal organ of involvement, grade of tumor or interferon therapy could not be statistically analyzed as subsets because of the small number of patients. However, all the three living patients had locally invasive disease prior to surgery and their histological characters are not different from the other patient population. One among the three patients received interferon therapy. To summarize, there are no notable differences in patient /disease/ treatment characters, in those who had longer survival.

When palliation of symptoms was assessed, it was found that the majority (10 out of 11) of patients who had pain as their major preoperative complaint had substantial reduction in pain. Hematuria as the chief symptom was relieved in all patients who underwent resection (four out of four). Needless to say, palpable abdominal mass, which was a cause for considerable alarm in many patients, disappeared in all cases when tumor was excised. Furthermore, of the 13 patients who underwent resection and subsequently died, only two patients experienced symptoms due to local recurrence prior to death.

## DISCUSSION

According to the literature, RCC will have extended into adjacent organs in around 10% of patients at diagnosis.[11,12] In spite of this, locally invasive RCC has received relatively little attention in the literature and historically was an autopsy finding usually associated with distant metastases.[4]

Computed tomography or MRI studies can demonstrate adjacent organ involvement preoperatively, but adjacent organ involvement is often difficult to assess.[13] Also, imaging methods have a tendency to overestimate the degree of invasion and unless obviously inoperable, patients should not be denied the option of exploration.[14]

In Robson's series,[12] 9% of patients had invasion of adjacent organs and 20% of these survived three years. DeKernion and associates reported a less than 5% three-year survival rate.[10] Gittes reported negligible long-term survival with RCC involving liver or bowel.[6] In Bennet's series of three patients with RCC with direct hepatic extension treated by en bloc partial hepatectomy, two experienced rapid disease progression and died, but one patient had worthwhile disease-free status.[15] In a Japanese study of seven patients,[5] a mean survival time of 14.2 months was reported with one patient surviving six years, this series having one of the highest reported survival rates for locally invasive RCC. An overall 16% five-year survival for TNM Stage IV disease was reported by Guinan *et al.*[16] and on multivariate analysis only tumor size and performance of nephrectomy were found to significantly affect survival in these patients.

En bloc resection of the tumor along with all involved organs is the procedure of choice in the management of RCC involving adjacent organs. In DeKernion's series,[10] barely 12% of patients who underwent incomplete excision of locally invasive RCC were alive at one year. In fact, patients with incomplete tumor excision because of locally extensive disease have a prognosis that is poorer than even those with distant metastases and good local control.[17] In our study, both the patients who did not undergo tumor extirpation experienced rapid disease progression and died within four months of surgery.

The other important end-point in treating patients with locally invasive RCC is amelioration of symptoms. These tumors are usually large and the patient is symptomatic. Many patients have pain either due to invasion of parietal muscles or nerves or simply because of the dragging pain associated with a large mass. Uncontrolled hematuria is another symptom which can be totally controlled with surgery. It is worth operating on these patients who experience considerable relief from their symptoms for several months following surgery. Also, we have found that a significant majority of patients remain free of local symptoms until the time of death, thus providing effective palliation.

## CONCLUSION

Surgery should not be precluded in patients with locally advanced renal cell carcinoma, especially in the presence of symptoms. Most patients experience significant and durable improvement of their symptoms following extirpative surgery. Surgery is the only treatment offering the potential for long-term survival, even though the proportion of long-term survivors is small.
